# A Mathematical Framework for Protein Structure Comparison

**DOI:** 10.1371/journal.pcbi.1001075

**Published:** 2011-02-03

**Authors:** Wei Liu, Anuj Srivastava, Jinfeng Zhang

**Affiliations:** Department of Statistics, Florida State University, Tallahassee, Florida, United States of America; Fox Chase Cancer Center, United States of America

## Abstract

Comparison of protein structures is important for revealing the evolutionary relationship among proteins, predicting protein functions and predicting protein structures. Many methods have been developed in the past to align two or multiple protein structures. Despite the importance of this problem, rigorous mathematical or statistical frameworks have seldom been pursued for general protein structure comparison. One notable issue in this field is that with many different distances used to measure the similarity between protein structures, none of them are proper distances when protein structures of different sequences are compared. Statistical approaches based on those non-proper distances or similarity scores as random variables are thus not mathematically rigorous. In this work, we develop a mathematical framework for protein structure comparison by treating protein structures as three-dimensional curves. Using an elastic Riemannian metric on spaces of curves, geodesic distance, a proper distance on spaces of curves, can be computed for any two protein structures. In this framework, protein structures can be treated as random variables on the shape manifold, and means and covariance can be computed for populations of protein structures. Furthermore, these moments can be used to build Gaussian-type probability distributions of protein structures for use in hypothesis testing. The covariance of a population of protein structures can reveal the population-specific variations and be helpful in improving structure classification. With curves representing protein structures, the matching is performed using elastic shape analysis of curves, which can effectively model conformational changes and insertions/deletions. We show that our method performs comparably with commonly used methods in protein structure classification on a large manually annotated data set.

## Author Summary

Protein structure comparison is important for understanding the evolutionary relationships among proteins, predicting protein functions, and predicting protein structures. Despite its importance, there have been no rigorous mathematical or statistical frameworks for protein structure comparison. One notable issue in this field is that with many different similarity measures used in comparing protein structures, none of them are proper distances when protein structures of different sequences are compared. In this study, we develop a mathematical framework for protein structure comparison by treating protein structures as three dimensional curves. A formal distance, geodesic distance, can be computed for any two protein structures. In this framework, population-specific variations within protein families can be characterized through building probability distributions for structures of protein families. The mean and covariance computed from groups of protein structures can also help to improve the classifications of protein structures. With curves representing protein structures, the matching is performed using elastic shape analysis of curves, which can effectively model conformational changes and insertions/deletions.

## Introduction

Comparison of protein structures (or structure alignment) is an important tool for understanding the evolutionary relationships between proteins, predicting protein structures and predicting protein functions [1], [2]. In annotating functions of new proteins, such as those solved in structural genomics projects, sequence alignment methods may not be sufficient to identify functionally related proteins when the sequence identities between the query protein and its related proteins are low (i.e. lower than 20%) [3]. Comparing their structures provides an effective means of annotating protein functions based on the structural similarity of proteins since homologous proteins are more conserved in their structures than sequences [4]. To organize proteins by the similarity of their backbone structures, databases, such as SCOP [5], [6], CATH [7], [8] and FSSP [9] were built for all proteins of known structures in the protein data bank (PDB) [10] by manual annotation [5], [6], automatic classification [9] or combination of the two [7], [8]. As the structure information is increasing at an accelerated speed, human annotations have become more time and resource consuming. Automatic structure alignment methods developed in the past [11]–[36] can be largely divided into several categories according to the specific similarity metrics (distances) they aim to optimize to achieve the best alignment. The particular metric used reflects the emphasis of the method on what constitutes a good alignment between two structures. When using the same similarity measures, methods differ by how they achieve the optimal solution through various search algorithms. Several studies have been performed to comprehensively compare different structure alignment methods [37]–[39]. The conclusions from these studies are that there is still room for improvement in structure alignment and there is no common standard for assessing the quality of alignment. Different criteria tend to rank methods differently and for a particular purpose one method may work better than the others. But, in general, no one method works better than others for all purposes. Despite extensive studies in the past, structure alignment, especially flexible structural alignment (i.e. one of the structures has undergone some conformational changes), continues to be a very challenging problem [37]–[39]. Another problem in structure alignment is to assess the statistical significance of the similarity between two protein structures. This problem is partly due to the lack of a proper metric for measuring the distance between two protein structures [40]. The root-mean-square-deviation (RMSD) of aligned parts between two structures has been commonly used to measure the similarity between pairs of protein structures after they are superposed. However, RMSD is not a proper distance when different sets of atoms are used to align different pairs of structures. Other similarity scores have also been used to derive statistical methods for evaluating significance of similarities. They suffer from the same drawback of RMSD as not being proper distances. In addition, a problem with many of the current metrics is that the best alignment between two structures, corresponding to the minimum value of the alignment metric used, cannot be obtained easily. Heuristic methods are often used to search the alignment space to find the best alignment, producing approximate minimum distances with possible biases.

A previous study aiming to develop a statistical framework for structure alignment [41] inferred the probability distribution of similarities of unrelated proteins by performing large scale alignment of protein structures. The resulting pair-wise alignment scores are then fitted to an extreme value distribution. It has also been pointed out that in these frameworks, the commonly used metric RMSD does not lead to as reliable a measure of structural significance compared to some “less proper” distances such as the alignment scores [41]. This raised some concerns over these statistical frameworks.

In this study, we develop a mathematical framework for protein structure comparison using a formal distance, a geodesic distance based on a particular Riemannian metric. Geodesic distances in elastic shape analysis have been used widely in shape analysis in computer vision [42]–[45]. An advantage of this approach is that the dynamic-programming algorithm can efficiently compute the optimal alignment between two protein structures. In this framework, we consider protein backbones as continuous three-dimensional curves. The alignment of two protein structures then becomes alignment of the two curves derived from the two backbone structures. Curves can bend and stretch readily during alignment so that the flexibility of and variations among protein structures can be adequately accounted for. Our goal is to develop a *comprehensive framework for statistical analysis of protein structures*. This framework can: (1) Generate optimal matching of protein backbone structures using shape information, where a formal distance, geodesic distance, is computed as a measure of the dissimilarity between shapes of any two protein structures. The optimal matching of two structures, computed by dynamic programming algorithm, gives the minimum distance among all possible matchings of two structures. (2) Compute statistical averages of a collection of structures using geodesics and geodesic distances. Such tools can be further advanced to define statistical models for capturing variations in protein conformations and for classifying future discoveries into pre-determined classes. That is, one can generate mean and covariance associated with a set of protein structures and characterize the central behavior of a population. (3) Generate optimal deformation of one backbone into another using geodesic path in the shape space. This work is an extension of a recent framework for comparing shapes of curves in Euclidean spaces, called the *elastic shape analysis* [42], [43], [46].

The rest of this paper is organized as follows. We first describe the mathematical framework that is behind our approach to protein structure comparison. We then use some examples to illustrate this method in pair-wise structure alignment and in computing mean and covariance of a group of protein structures. We further demonstrate the performance of our method using a large-scale classification of proteins in SCOP database and compare our performance with some commonly-used methods. Finally, we conclude the paper with discussions.

## Methods

### The mathematical framework

We treat the backbone structure of a protein as a parameterized curve in *R* ^3^. Given any two such parameterized curves, we desire a framework that can quantify the differences in shapes of these two curves. Since the comparisons involve shapes of proteins, the resulting quantifications should not depend on the rigid motions and parameterizations of these curves. We will use a Riemannian framework for this task and the basic idea in this approach is the following. We represent each parameterized curve by a special function called the *square root velocity function* (SRVF) and restrict to the manifold of such functions under the desired constraints. In order to compare shapes of curves, we have to remove all the shape-preserving transformations from this representation. This is done using an algebraic technique – we form a quotient space of the original manifold with respect to these shape-preserving transformation groups. In the resulting quotient space, called the *shape space of elastic curves*, one can perform statistical analysis of curves as if they are random variables. One can compare, match, and deform one curve into another, or compute averages and covariances of curve populations, and perform hypothesis testing and classification of curves according to their shapes. The mathematical details are provided next.

#### Elastic representation of protein structures

To derive a curve from a protein structure, we take the sequence of 3D coordinates of the backbone atoms N, CA and C from the PDB [10] file and treat them as the coordinates *β*(*t_i_*) = [*β*_1_(*t_i_*) *β*_2_(*t_i_*) *β*_3_(*t_i_*)], *i* = 1,2,…,*n*, for *n* atoms. We use *t_i_* = *i*/*n* so that the parameter lies between [0,1]. These points become samples along the curve and we can compute *β*(*t*) for any *t* in [0,1] using interpolation. Note that in our method it is not necessary for the curve to be arc-length parameterized, i.e. the distances between *β* (*t_i_*)s need not be same. Since we optimize over all re-parameterizations of the curves, any initial parameterization read from the PDB file is just fine.

Let the parameterized curve in *R* ^3^ derived from the backbone structure of a protein be denoted as *β* : [0, 1]*→R* ^3^. In order to analyze its shape, we will represent *β* by its square-root velocity function: 
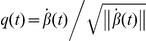
 in *R* ^3^, where 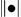 is the standard Euclidean norm in *R* ^3^. The SRVF *q* includes both the instantaneous speed (
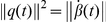
) and direction (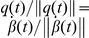) of curve *β* at time *t*. The use of the time derivative makes SRVF invariant to any translation of curve *β*. Conversely, one can reconstruct the curve *β* from *q* up to a translation. In order for the shape analysis to be invariant to scales, we rescale each curve to length 1. With a slight abuse of notation, we will denote the rescaled curves by *β*. Since 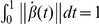, we have: 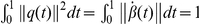. In other words, the 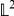 norm of the SRVF is one. Restricting to the curves of interest, represented by their SRVFs, we obtain the set
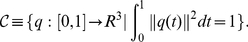
(1)

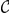
 is called the *preshape space* and is the set of all SRVFs representing parameterized curves in *R* ^3^ of length one. It is actually a unit sphere in the Hilbert space 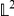.

We have mentioned four shape-preserving transformations – translation, scale, rotation, and re-parameterization. Of these, we have already eliminated the first two from the representations, but the other two remain. Curves that are within a rotation and/or a re-parameterization of each other result in different elements of 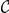 despite having the same shape. The removal of the remaining two transformations is performed algebraically as follows. Let *SO*(3) be the group of 3×3 rotation matrices and 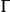 be the group of all re-parameterizations (they are actually positive diffeomorphisms of the interval 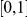). For a curve *β*, a rotation 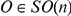 and a re-parameterization 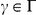, the transformed curve is given by 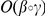. The SRVF of the transformed curve is given by 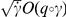. In order to unify all elements in 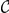 that denote the same shape we define equivalence classes of the type: 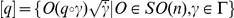. Each such class [*q*] is associated with a shape uniquely and vice versa. The set of all these equivalence classes is called the *shape space* 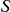. Mathematically, it is a quotient space of the preshape space: 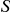 = 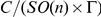.

#### Elastic metric

When we deform one curve into another we are actually generating a continuous sequence of curves, or a path in the curve space, and a natural question is how long that path is. The length of this path also quantifies the amount of deformation in going from one curve to the other. The question changes to: what should be the metric to measure this path length. An elastic metric is a metric that measures the amount of bending and stretching between successive curves along the path and adds them up for the full path. Mio et al. [44] defined a family of elastic metrics depending upon how much relative weight is attached to bending and stretching. Joshi et al. [42], and more recently Srivastava et al. [45], proposed the SRVF that has the special property that under this representation, the elastic metric turns into (using a change of variables) the standard 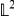 metric. That is, one can alternatively compute the path lengths, or the sizes of deformations between curves, using the cumulative 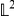 norms of the differences between successive curves along the paths in the SRVF space. This turns out to be much simpler and a very effective strategy for comparing shapes of curves, by finding the paths with the least amounts of deformations between them, where the amount of deformation is measured by an elastic metric.

Let 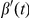 be the velocity function along a curve *β*(*t*) and let 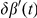 a perturbation of that velocity function. A Riemannian metric is a metric that measures the norm of the perturbation ∥
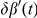
∥. If we represent the vector 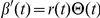, where *r*(*t*) is the magnitude of 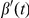 and Θ(*t*) is the direction of 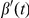. Rather than computing ∥
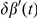
∥, one can separately compute the norms of these two components and that defines an elastic metric:
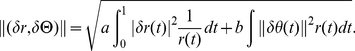
(2)The first term in the square-root is the measurement of stretching and the second term is a measurement of bending in 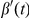 introduced by 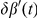. Depending on the values of *a* and *b*, one gets a whole class of metrics called the elastic metrics. This metric is rather complicated to implement and use in shape analysis of curves. It was shown by Joshi et al [42] that if we represent a curve *β* by its SRVF *q*, then the corresponding norm on *δq*(*t*) is actually the 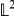 norm. That is,
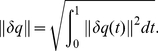
(3)Thus, the use of SRVF greatly simplifies the use of the elastic metric.

The so-called preshape space 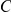 is a nonlinear manifold because it is a unit sphere. We cannot perform calculus on this space as if it is a vector space. Operations such as addition, subtraction, and multiplication are not available on nonlinear spaces. This means that we cannot use standard techniques in multivariate statistics for inferences on 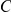 and 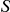. This problem is solved by mapping points from the manifold to a plane that is tangent to the manifold (at a certain point), statistics are computed in the tangent space, and then mapped back to the manifold. Since the mapping back and forth is unique (under some appropriate constraints), such computations are well defined and the estimates are consistent. *Tμ*(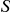) is the notation for all the functions that are tangent to the manifold 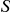 at the point *μ*. In case of spherical manifolds, it is easy to visualize what the tangent spaces are.

#### Shape comparisons and averaging

Once we have a Riemannian manifold, we can compute distances between points in that manifold. For any two points, the distance between them is given by the length of the shortest path (called a *geodesic*) connecting them in that manifold. An interesting feature of this framework is that it not only provides a distance between two protein structures, thus quantifying differences between their shapes, but also a geodesic path between them in 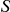. This path has the interpretation that it provides the optimal deformation of one shape into another. The geodesics are actually computed using the differential geometry of the underlying space 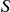. Consider two curves *β*_1_ and *β*_2_, represented by their SRVFs *q*_1_ and *q*_2_. In order to compute geodesics between their equivalence classes [*q*_1_] and [*q*_2_], we fix *q*_1_ and find the optimal rotation and re-parameterization of *q*_2_ to solve:
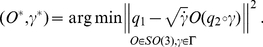
(4)The optimization over rotation is straightforward, using SVD, but the optimization over the re-parameterization requires a dynamic programming algorithm. Please note that the optimal γ* is the *matching function* between the two backbone structures. Define 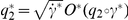 and compute a geodesic path between *q*_1_ and 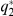 in 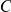. Since 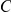 is a sphere, the geodesic between any two points is given by a great circle whose equation is:
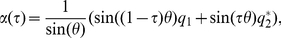
(5)where *α* is a geodesic path between the given two shapes such that it is in [*q*_1_] at *τ* = 0 and in [*q*_2_] at *τ* = 1. Here 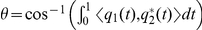 is the distance between the two equivalence classes in 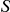, i.e. *d*([*q*_1_], [*q*_2_]) = *θ*. This *θ* is a **proper distance** in the shape space as it satisfies all the three properties of a distance function, including the triangle inequality. In practice, the matching function *γ* is represented as a set of discrete values along its domain. If we use *n* point to represent *γ*, then we can represent *γ* in a computer using the vector [*γ* (0), *γ* (1/*n* ), *γ* (2/*n* ), *γ* (3/*n* ),… *γ* (*n*−1/*n*), *γ* (1)]. To apply a re-parameterization *γ* to a curve *β*, we compute the new curve *β* (*γ* (*i*/*n*)), for *i* = 1,2,..,*n*. Since *β* values are available for only discrete *t*'s, we interpolate in between the given values to obtain the new *β*, which is done using bilinear interpolation.

**Figure 1:**
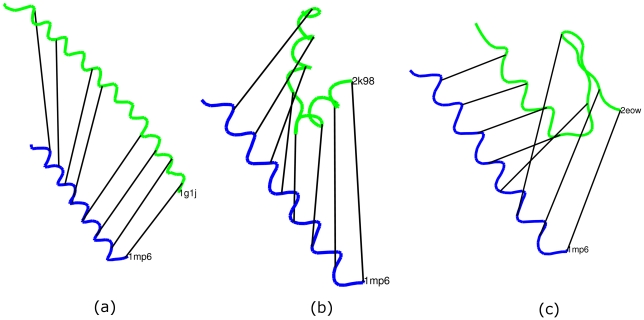
Examples of elastic geodesics. a) Elastic matching between protein 1MP6 and 1G1J. b) Elastic matching between protein 1MP6 and 2K98. c) Matching between protein 1MP6 and 2EOW.

Fig 1 shows three simple examples of elastic matching using four small proteins (PDB IDs: 1MP6, 1G1J, 2K98 and 2EOW). In Fig 1a, protein 1MP6, a protein with a single helix, is matched to protein 1G1J, which also has a single helix but longer and bent in the middle. We can see that the helix of 1MP6 is matched to two relatively straight parts of the helix in 1G1J and the bent region is skipped. Fig 1b shows the matching between 1MP6 with 2K98, a protein with a helix-turn-helix structure. The helix in 1MP6 is matched to both helices in 2K98. This kind of matching cannot be achieved with rigid alignment methods. In Fig 1c, we match protein 1MP6 to protein 2EOW (residue 12–37) with an alpha helix and a beta sheet. We can see that a small portion of the chain at the end of 1MP6 is stretched to match with the sheet in 2EOW. Since the change of shape in this case is more than the matching in Fig 1a and 1b, the distance between 1MP6 and 2EOW (0.943) is larger than that of 1MP6 and 1G1J (0.668) and that of 1MP6 and 2K98 (0.895). Secondary structure information is not used in these matchings. It is possible to match only alpha helices with alpha helices and beta sheets with beta sheets when additional secondary structure information is incorporated (see Discussion). It is worth mentioning that the matching of two conformations of the same protein (such as two NMR models of the same protein) produced by elastic shape analysis does not necessarily have the corresponding residues of the protein matched. This is so because matching of the corresponding residues is not a constraint in calculating the distance and thus may not be satisfied.

**Figure 2:**
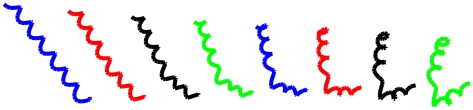
Geodesic path between protein 1MP6, the left-most structure, and protein 2K98, the right-most structure.

In Fig 2 we show the geodesic path from 1MP6 to 2K98. We can see that 1MP6, the left-most structure, transforms its shape to 2K98, the right-most structure, by bending the middle portion of the straight helical structure. Under the current elastic matching approach, there may not be a physically meaningful explanation of the geodesic path since we allow both bending and stretching of the curves. But with some restrictions on how the curves can be manipulated, geodesic paths may shed some light on the conformational changes or dynamics of protein structures when different conformations of the same protein are compared.

**Figure 3:**
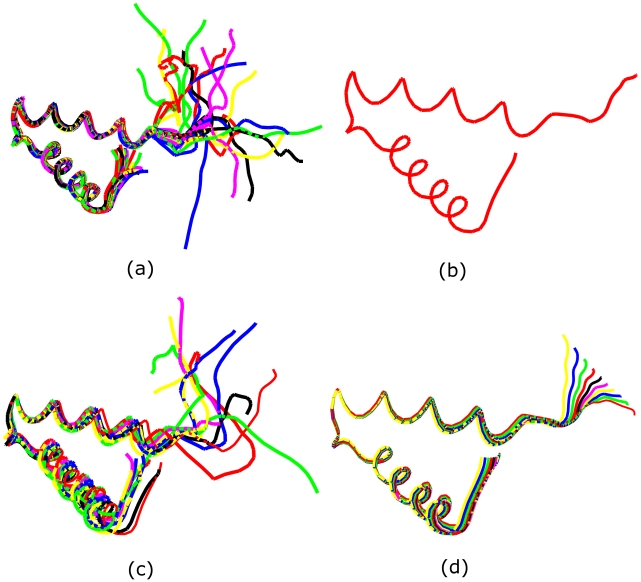
Mean structure and sampled structures. a) 2JVD NMR structures. b) The mean structure of multiple 2JVD NMR structures. c) Samples from the probability distribution. d) Samples on the largest variation direction.

Proteins are flexible molecules and conformational dynamics is important for protein functions [47], [48]. It has been proposed that protein structures should be characterized as ensembles of structures instead of single structures as seen in X-ray crystallography [49], [50]. In NMR structure determination, multiple models are often used to describe protein structures in solution. One natural, and statistically appropriate, representation of an ensemble of structures would be a probability distribution, where the mean of the distribution is the mean structure of the ensemble and covariances characterize the structure variations at different parts of the structure. This representation allows one to compute statistics of shapes as if they are random variables. Different probability distributions, representing different protein families, can be compared using standard testing. Hypothesis testing on whether a protein structure belongs to a known family of proteins can be performed using likelihood-ratio tests. Structure variations within a protein family can also be studied under this mathematical framework. For example, given a few sample shapes from a population, this method can produce their average shape in a principled manner. Let *β*_1_, *β*_2_, *…*, *β_n_* be a given set of structures, represented by their SRVFs *q*_1_, *q*_2_, *…*, *q_n_*. Define their mean shape as the quantity:
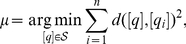
(6)where *d*([*q*],[*q_i_*]) is the distance between *q* and *q_i_*. The actual minimizer is found using an iterated gradient-approach that is not repeated here due to the lack of space but has been presented in many papers earlier (see e.g. [42]). Consider the 20 NMR structures of protein 2JVD obtained from the PDB (shown in Fig 3a). We have calculated the mean structure of these 20 NMR structures, and the result is shown in Fig 3b. Mean shapes of protein structure families/classes can be very useful in automatic classifications of new protein structures. For example, they can serve as filters to quickly narrow down the list of more likely protein families, which can then be studied in more details. In addition to the first central moment, i.e. sample means, we can also calculate covariances for a group of structures and even build a Gaussian-type probability distribution for this group by considering the available structures as a sample from the underlying distribution.

#### Covariance and its use in structure classification

One unique feature of the framework is its ability to calculate covariances for populations of protein structures. Covariances can reveal the population-specific variation among a group of protein structures and be used in classification of protein structures. From the covariances we can identify the directions with the largest variation within a group of protein structures. To define sample covariance we first approximate the shape space 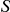 in a neighborhood of 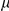 by a flat space *T_μ_*(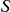). Then, each of the observed structures, or rather its SRVF, is transferred to the flat space *T_μ_*(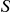) using the mapping:
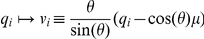
These *v_i_*s are simply the directions of the geodesics from the mean *μ* to *q_i_*s. We can compute the standard sample covariance matrix *K* of *v_i_*s and take its singular value decomposition *K* = *UΣU^t^*. Here *Σ* is a diagonal matrix of singular values (*σ*_1_, *σ*_2_, *σ*_3_…) and *U* contains the corresponding singular vectors. If the singular values are arranged in a decreasing order, the first few, say *k*, columns of *U* represent the directions of major variation, or the principal components, in the underlying population. If we let *z*_1_, *z*_2_, … *z_k_* be independent standard normal random variables, we can define a multivariate normal density on the direction *v* according to:
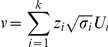
Then, this random direction can be converted into the SRVF of a random shape using the mapping
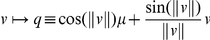
which can be further converted into a shape using integration:
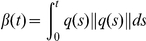
This defines a formal probability model on the shape space 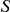 and one can sample random structures from it using the steps outlined above. Fig 3c shows 10 randomly sampled structures from such distribution with parameters estimated from the given structures.

**Figure 4:**
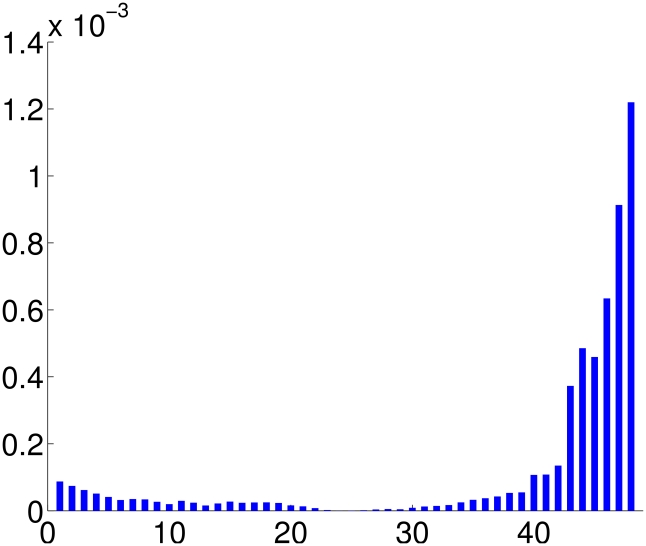
Flexibilities at each residue of 2JVD. X-axis is the indices of residues and y-axis is the variations of the residues.

If we set 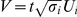 for some *i*, we can study the resulting shape changes in the direction of *U_i_*. By computing the shapes for a range of *t* from −2 to 2, we can see the shape variability in the structures for that direction. For example, in Fig 3d, we sample 10 structures along the largest variation direction *U*_1_. To display the rigid superposition of the structures we translate them so that their centres of mass coincide with that of the mean structure. The rotations were obtained through optimally matching the SRVFs of these structures to the mean structure. The variation can be decomposed to residue level and the flexibility (structure variation) of each residue can be analyzed. To obtain the variance for each residue, we sampled randomly 10 structures and align them with the mean shape. The distances of each residue of sampled structures with that of mean shape can then be calculated and used to compute variance of that particular residue. In Fig 4, we plot the variance of each residue for 2JVD. We can see clearly that those residues at the C terminal have much larger variation, which is consistent with observation from the multiple NMR structures.

**Figure 5:**
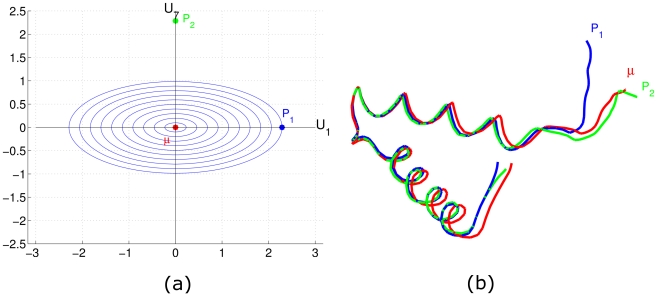
Covariance in structure classification. a) Illustration of two directions with different variations. b) Two structures sampled on the two directions of different variability but with similar distance to the mean shape.

To illustrate how we can use the covariance for structure classification, we sampled two random structures from the distribution built using multiple NMR structures of protein 2JVD with the same geodesic distance to the mean structure, but along two different directions. The probabilities of the two structures under the calculated distribution are 0.0429 and 0.0048, respectively. Although they have the same distance to the mean structure, their probabilities are quite different. This is so because structure 1 lies in the direction with largest variability in the population and structure 2 lies in the direction with much smaller variability, as shown in Fig 5a. Fig 5b shows the superposition of the mean structure, structure 1 and structure 2. This can be a common scenario in structure classification in practice where by chance two proteins may be more similar in the parts that are not conserved within their own families, but less similar in the conserved parts. That will give a relatively small distance for the two proteins although they are not in the same family. Classification of such proteins can be improved using covariance structures of their corresponding families. In the sequence alignment, profiles can be built for families of sequences to achieve better sensitivity and accuracy [51]. However, profiles for protein structures are much harder to build. The current framework can readily account for the family-specific variations and use them in structure classifications.

## Results

In this section, we present the performance of our method on a large scale protein structure classification using structures from SCOP database and compare our results with CE [12] and Matt [25]. We selected a subset of non-homologous proteins from SCOP database with pair-wise sequence identity smaller than 40% from the four largest classes (with at least 5 members) at top level of SCOP hierarchy (all alpha, all beta, alpha/beta and alpha+beta). Classes at the bottom level (family level) with less than 20 members are ignored. This gave us a set of 1579 proteins in total. We calculated the pair-wise geodesic distances among these protein structures and clustered them into different classes. Hierarchical clustering is done using the cluster function in Matlab and average linkage is used to calculate distances among clusters. When number of clusters, *n*, is provided, the hierarchical clustering results can be easily divided into *n* classes. For CE and Matt, we used (1−score/score_max) as the distance, where score is either z-score provided by CE or a matching score provided by Matt, and score_max is the maximum z-score (for CE) or maximum matching score (for Matt) among all pairwise scores. Using the scores directly gave worse performances. We then used random index (RI) as a criterion to evaluate the accuracy of our classification. RI measures the percentage of correct decisions by looking at all pair-wise decisions, which is the ratio ((TP+TN)/(TP+TN+FP+FN)), where TP is true positive for a pair of proteins, which are in the same class in SCOP and classified into the same class, and TN (True Negative), FP (False Positive), FN (False Negative) are defined similarly. In Table 1, we compare the performance of our method with CE and Matt. To show how the methods perform for different types of proteins classified at the top level, we also show the results for these classes. We can see from Table 1 that our method, without using any secondary structure information, is comparable with CE and Matt overall. It is interesting that these methods have quite different performances for some protein classes.

**Table 1:** Performance comparison of elastic shape analysis (ESA) with Combinatorial Extension (CE) and Matt.

	ESA		CE		Matt	
	TP/TN/FP/FN	RI	TP/TN/FP/FN	RI	TP/TN/FP/FN	RI
A 229/9	2417/16880/6308/501	0.7392	2778/18721/4467/140	0.8235	2728/5643/17545/190	0.3207
B 516/13	10786/84675/35621/1788	0.7185	12193/91395/28901/381	0.7796	12137/97311/22985/437	0.8237
C 516/17	6624/98811/24907/2528	0.7935	8075/82681/41037/1077	0.6830	8827/106865/16853/325	0.8707
D 292/8	5728/34756/1324/678	0.9529	6361/30684/5396/45	0.8719	6406/36080/0/0	1
Total 1579/48	24409/1117980/96476/6966	0.9170	29431/1125707/88749/1944	0.9272	30042/1069181/145275/1333	0.8823

TP: true positive, TN: true negative, FP: false positive, FN: false negative. RI: random index scores.

**Figure 6:**
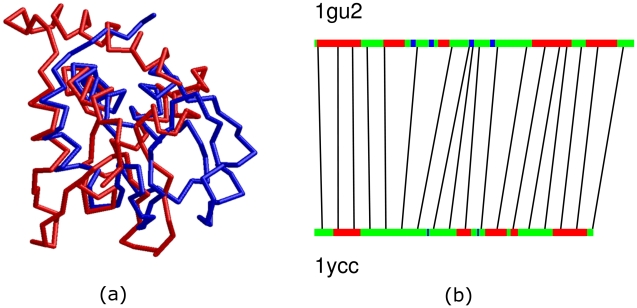
Example of structure alignment by ESA. a) Rigid superposition of 1ycc and 1gu2. b) Matching of points along 1gu2 and 1ycc by elastic shape analysis. The red regions label helices, green regions label strands, and blue regions label coils.

An example that illustrates the strength of our method is protein pair 1ycc and 1gu2, which have a small geodesic distance (0.84) and are correctly classified into the same family by our method. For these two proteins, CE gives a small z-score (2.6) and classifies them into different classes. DaliLite and Mammoth give z-scores of 3.2 and 1.6, respectively (small scores imply large distances). Matt, a method for flexible protein alignment, gives a *p*-value of 0.03 showing the two proteins have statistically significant similarities. The rigid alignment of the two proteins by Mammoth is shown in the left panel of Fig 6 and matching of the two proteins by ESA is shown in the right of Fig 6. One can see that a rigid alignment aligns the two proteins rather poorly. On the other hand, flexible alignment methods like Matt and ours can match them quite well.

Finally we compared the running time of our method with several other methods. Table 2 shows the comparison of running time of CE, Matt, MUSTANG and ESA on three pairs of proteins with around 100, 200 and 300 residues, respectively. All the programs were run using the same computer.

**Table 2:** Running time comparison of different methods.

	∼100 residues	∼200 residues	∼300 residues
CE	1.3	3.0	5.1
Matt	0.16	2.30	2.1
Mustang	1.2	2.6	15
ESA	0.74	1.04	1.54

All the times are in seconds.

## Discussion

In summary, we have developed a mathematical framework for protein structure comparison based on elastic shape analysis, a method originally developed in the field of computer vision and image analysis. Under this framework, protein structures are compared as three dimensional elastic curves and can be treated as random variables for statistical analysis. Mean and covariance of a group of protein structures can be computed. Probability distributions can be built for a population of protein structures and hypothesis testing can be conducted for a protein structure against a known protein family/class. Although protein structures have been studies for many years and many computational methods have been developed for protein structure comparison, as far as we know, this is the first rigorous mathematical framework that can address the above computations.

It is worth mentioning that although we consider protein structures as three dimensional curves in this study and ignore the sequence and local structure features (such as secondary structures), the framework can readily incorporate amino acid sequence or secondary structure information. Such additional information can be very helpful to achieve better alignment. For example, secondary structure information has been used by many structure alignment methods since secondary structure type is the major feature used in manual structure classification. To incorporate such auxiliary information we can construct continuous auxiliary functions along the curves, derived from the additional information. The matching and deformations can then be performed using the higher dimensional composite curves that are formed by concatenating the geometric and the auxiliary coordinates. The distances obtained are still proper distances on the higher dimensional space. In this matching, one needs to adjust the relative magnitude (weight) of the geometric and auxiliary coordinates, which can be problem dependent. With secondary structure type as auxiliary function, we can force protein fragments with the same secondary structure type to match with each other by giving a larger weight on the auxiliary secondary structure information, which may further improve the accuracy of structure classification. When using sequence as auxiliary information, one can perform alignment on both structure and sequence space by using an amino acid substitution matrix (for instance, BLOSUM62 matrix) as the distance measure for amino acid residues along the chains. One can also force all corresponding residues to match with each other when comparing two protein structures with the same sequence. The geodesic path (deformation from one structure to another) generated using such constraint may then have a more natural physical interpretation.

In this study we focused mainly on pairwise protein structure comparison and studying the basic properties of a population of structures such as means and covariances. The framework can also be applied to study multiple structure comparison (multiple structure alignment) and provide an alignment of multiple structures if it is desirable. To do so, we can calculate the mean structure of the multiple structures and align each structure to the mean structure. The mathematical framework also provides principled ways to deal with more complex situations. For example, in the troublesome case that there is one or more structures that are very different from the rest of the structures to be aligned, outliers can be detected based on the mean and covariance structure of the population.

In constructing a probability distribution from a group of structures, we chose the tangent space of our shape space and assumed Gaussian distribution on this space. The shape spaces (ours and most other formally defined shape spaces) are highly nonlinear manifolds and it is difficult to build distributions on them directly. On the other hand, it is a very common practice to impose probability distributions on the tangent space since they are linear (vector) spaces. The mapping between a tangent space and the manifold can be made a bijection by putting some appropriate constraints on the tangent space. As for the choice of Gaussian distribution, we have not validated it on the tangent spaces of our shape space. Our goal in this study is to demonstrate the computation of the second moment for observed shapes and to suggest the simplest probability model that captures the first two moments, i.e. a multivariate normal. One can easily extend this framework to include mixtures of Gaussian models [52] or even generalized Gaussian models and we expect them to better match the observed variability of the protein structures. These extensions can be explored in future studies.

Since we represent protein structures as curves, our method mainly deals with the type of structure comparison where sequence order of amino acid residues is relevant to the distances of structures (sequential structure alignment). In general, our method is not good at detecting related proteins whose differences are caused by changes such as domain swapping, or domain insertion/deletion. However, the method can be readily modified to compare circular permuted proteins [33] by linking the C-terminal and N-terminal ends of a protein (for example, using a straight line) and cutting the protein in the middle, preferably at residues linking domains or secondary structure fragments. To deal with domain insertion/deletion/swapping, we can use the algorithm in [33], where this problem is formulated as a mixed-integer programming problem, to select near optimal combination of fragments before calculating geodesic distances. If domain swapping or deletion/insertion can be detected or predicted (i.e. using sequence based methods), cuts and reconnections can also be done at corresponding positions to allow for even more flexible structure comparisons.
